# HEteronuclear Referencing for METRologic Isotope Calibration (HERMETRIC)

**DOI:** 10.1002/mrc.70020

**Published:** 2025-08-18

**Authors:** Bernd W. K. Diehl, Jakob V. Waldthausen, Yulia Monakhova

**Affiliations:** ^1^ Spectral Service AG Cologne Germany; ^2^ Faculty of Applied Sciences University of Applied Sciences Bonn‐Rhein‐Sieg Rheinbach Germany; ^3^ Department of Chemistry and Biotechnology University of Applied Sciences Aachen Jülich Germany; ^4^ Institute of Chemistry Saratov State University Saratov Russia

**Keywords:** analytical principles, complexometry, heteronuclear quantitative NMR spectroscopy, isotopic analysis, methods comparison, titration, weighing

## Abstract

This work presents the concept of heteronuclear referencing for metrologic isotope calibration (HERMETRIC) as an innovative approach to quantitative NMR (qNMR) spectroscopy. The aim is to establish a metrologically based fundamental understanding that goes beyond traditional homonuclear NMR methods. In comparison to established quantitative methods—such as weighing, titration, chromatography, and complexometry—it is demonstrated that qNMR, as a primary method, can determine absolute amounts of substance directly without external calibration. At the same time, heteronuclear quantification opens new perspectives by enabling the direct traceability of all active nuclei to a universal qNMR primary standard. The concepts are intended to encourage the further establishment of qNMR as a powerful instrument in analytical chemistry and its incorporation into pharmacopoeias and other official standard protocols.

## Graphical abstract

The graphical abstract shows a steelyard balance with the counter weight indicating the amount of deuterium as internal standard defined through D_2_O addition. The bowl of smarties represents the variety of NMR nuclei this principle was and can be applied to.

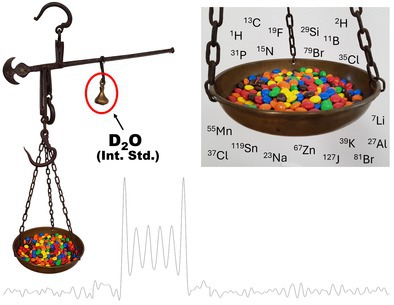

## 1 Introduction

The field of quantitative NMR (qNMR) spectroscopy has experienced continuous innovation in recent years [1, 2]. Many studies have traditionally focused on the analysis of individual substances—predominantly using ^1^H‐NMR, and occasionally ^19^F or ^31^P‐NMR [3, 4, 5, 6, 7] especially to analyze complex mixtures [8]. These nuclei are commonly used thanks to their 100% or nearly 100% natural abundance and the availability of suitable instruments. A variety of applications fall into the field of analysis of pharmaceuticals [9, 10, 11], bioactive natural products [12], or pesticides [13]. However, the application of qNMR often reveals a fundamental misunderstanding of the method, as many practitioners default to classical chromatographic approaches. In response, the aim of this study is to establish a new, metrologically founded basic understanding—enriched by insights from the philosophy of science, formal logic, and aesthetics. NMR spectroscopy, reflecting the inherent symmetry of the quantum mechanical world, essentially functions as a quantum mechanical balance.

Moreover, the strategy of investigating signals from all NMR‐active nuclei—and even some inactive—is not new. Since the early days of modern FT NMR spectroscopy, numerous influential works have ventured beyond conventional proton signals, exploring the entire spectrum of nuclei [14, 15, 16]. This rich historical background reflects the evolution of NMR spectroscopy, and while it is impossible to comprehensively cite all contributions, we extend our sincere gratitude to the pioneers who laid the groundwork for this field. Our goal is to thoroughly develop this fundamental qualitative knowledge from the perspective of complete quantitative analysis. Each idea discussed in this paper should be explored separately in detail; the advantages and disadvantages as well as practical application of each approach should be investigated.

Acronyms are a popular tool in NMR spectroscopy. With the acronym HERMETRIC, we intentionally embedded two key ideas. On one hand, “METRIC” reflects our project's focus on establishing a metrological approach to qNMR. On the other hand, the prefix “HERME” hints at the philosophical concept of “hermeneutic.” Derived from the Ancient Greek ἑρμηνεύειν (hermēneúein, meaning “to explain,” “to interpret,” or “to translate”), hermeneutics is the theory of text interpretation and understanding. According to its definition, hermeneutics considers that humans understand the world through symbols and shared language, attributing meaning not only to texts but also to all human creations. NMR spectra are our “text” and “creation”—a language not composed of varied scripts but of a universal image: the spectrum. Uncovering its inherent meaning is a hermeneutical task—a notion we extend into the realm of qNMR spectroscopy.

In antiquity and in the Middle Ages, hermeneutics served as both the science and art of interpretation. By combining the metrological aspect with this hermeneutic perspective, our approach offers a holistic view of qNMR. We propose that this dual interpretation can inspire the community of NMR spectroscopists, particularly those engaged in quantitative analysis, to embrace a broader conceptual framework that unites precise measurement with a deeper understanding of meaning.

## 2 Principles of qNMR Spectroscopy Compared With Other Methods

qNMR is capable of determining absolute amounts of substances without external calibration. Compared with other primary methods such as weighing, titration, and chromatography, qNMR offers direct traceability to physical constants and high precision. Moreover, qNMR enables the determination of absolute concentrations without comparative standards. Chromatography excels at separating complex mixtures, while qNMR allows for direct quantification without a separation process through chemical environment specific chemical shifts. A defined internal standard serves as an orthogonal method for determining the content of reference substances, similar to methods used in chromatographic analyses. In most of the applications in this study, qNMR measurements using an internal standard are discussed. In the following sections, the principles of qNMR as a quantitative method are discussed on this basis. Our approach does not overwrite standard quantification approaches in analytical chemistry (and in NMR spectroscopy in particular) such as external calibration or standard addition, but shows specific features of qNMR spectroscopy, which can be exploited for other practical applications of this technique.

### 2.1 qNMR Compared With Weighing

**FIGURE 1:**
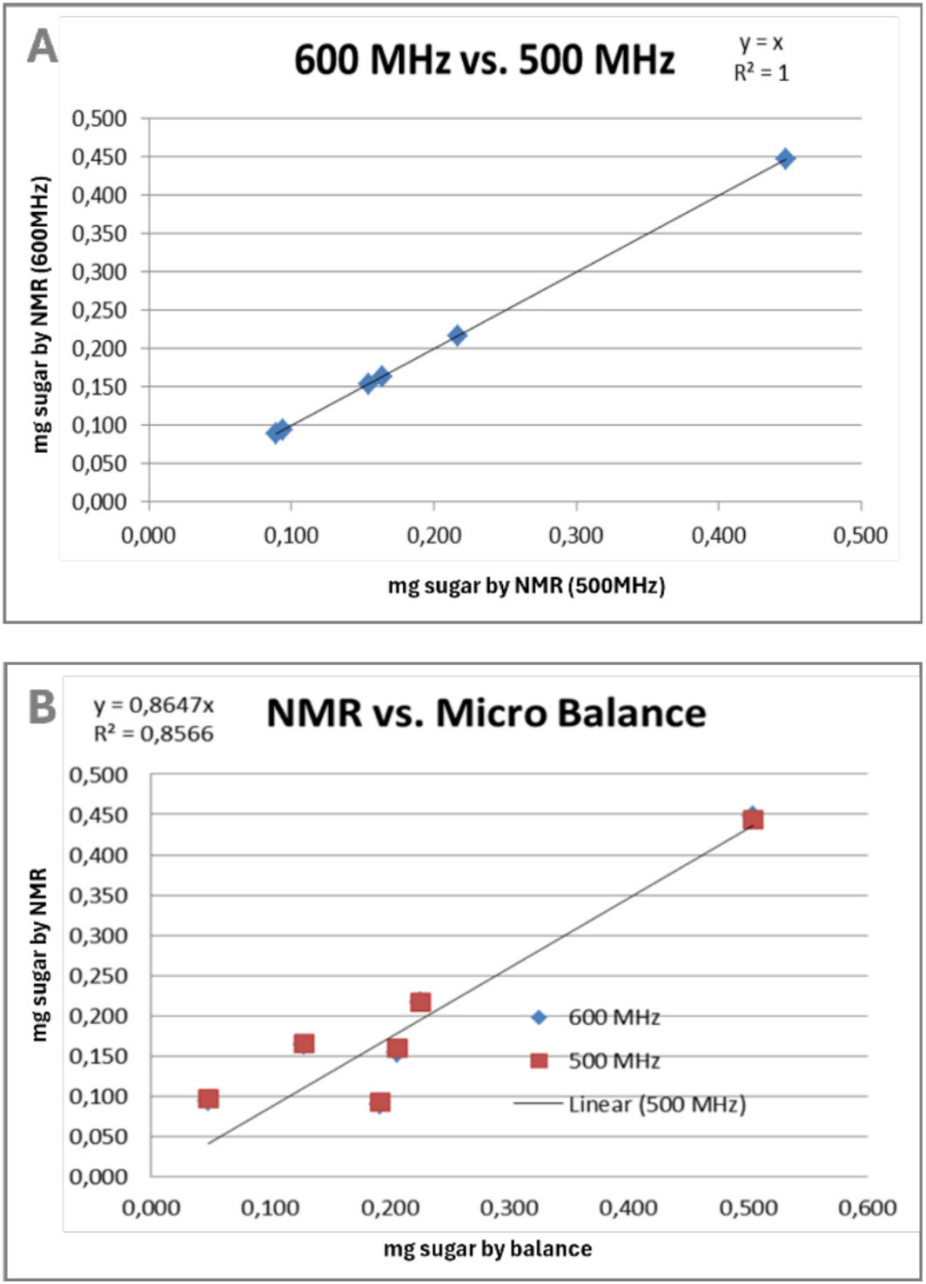
(A) Quantification of six sugar granules in milligrams shows identical values on different NMR devices of 600 MHz cryo and 500 MHz RT. External calibration, 10.53 mg sucrose in 1 mL (1103.85 mg) D_2_O, dilution 1/1, 1/2, 1/5, 1/10, 1/50. (B) Correlation of 600 and 500 MHz results with the initial weights. The weights of the sugar granules are between 0.1 and 0.5 mg and far under the balance's minimum weight of 2.5 mg.

Both qNMR and weighing are regarded as primary methods because they are based on natural law [17]. While weighing directly measures mass via gravitational force, qNMR is based on the quantitative detection of the number of atomic nuclei through signal intensities. A disadvantage of weighing is that it does not provide information on chemical purity, whereas qNMR can additionally determine both substance purity and molecular identity. The experimental series “The Weighing of the Unweighable” illustrates this: Individual sugar crystals were weighed using a precision balance, dissolved in exactly 1 mL of D_2_O, and analyzed by NMR. While the mass of a single sugar crystal lies below the minimum measurable threshold for weighing, qNMR measurements yielded consistent results even on different instruments (Figure 1).

### 2.2 Master and Servant in Methodology—Validation of Weighing Process Using qNMR

The above‐mentioned considerations raise the question of which method should dominate in calibration or validation. Secondary methods such as chromatography or titration are usually calibrated against a primary method, most commonly weighing. However, our results show that validating a qNMR method tests not only the balance's performance but also the entire process, including the technical competence of the laboratory personnel. QNMR should be used for monitoring the weighing processes in the laboratory [18]. For example, performing six qNMR measurements on an identical sample reflects the confidence interval of the NMR measurement, whereas conducting qNMR on six independently prepared samples provides insight into the quality of sample preparation. The NMR measurement can be regarded as largely independent of the laboratory personnel, which is not the case with weighing. Consequently, qNMR measurements can serve as performance verification of laboratory personnel under GMP conditions, with the relative standard deviation as the sole criterion (Figure S1).

Manual preprocessing and integration steps in qNMR can be substituted by automated qNMR quantification and results verification [19]. This routine was also introduced in a weighing testing exercise [20]. Laboratory personnel and study directors (*n* = 16) performed sixfold weighing of the binary mixture of butylated hydroxytoluene (BHT) and 1,2,4,5‐tetrachloro‐3‐nitrobenzene (TCNB). To evaluate the quality of data analysis, all spectra were evaluated manually by a qNMR expert and by using an in‐house developed automated routine. The results revealed that mean values are comparable and both evaluation approaches are free of systematic error. However, automated evaluation resulted in an approximately 20% increase in precision. The automated qNMR method significantly increases the throughput and precision of qNMR for routine measurements and proficiency testing.

### 2.3 The Swiss Army Knife of qNMR, the Nuclei Beyond ^1^H and ^13^C [21]

Until now, qNMR has been predominantly limited to homonuclear variants, in which the same nucleus is used both for analysis and as an internal standard (commonly proton, fluorine, or phosphorus). Our objective was to establish quantitative reference standards that cover a broader range of applications. With our “Swiss Army Knife” of qNMR, we combine all three nuclei within one molecule to provide a universal reference standard either simultaneously or sequentially. Through targeted syntheses, such standards can be optimized in terms of their physicochemical and spectroscopic properties. Important criteria include characteristic signals—not necessarily singlets—minimal interference with the substances under investigation, and a balanced ratio of nuclei to molecular weight. Additional aspects, such as relaxation times, solubility, stability, toxicity, purity, and ease of synthesis, also play a role—requirements that are best met by fluorinated aromatic phosphoric esters.

An extension of this approach enables heteronuclear standardization. In principle, the signal of any heteronuclei can be used for quantification, provided that the molar response of the nuclei being compared is known. Thus, sequential quantitative statements about multiple nuclei can be made, for example, through parallel analyses of proton and phosphorus NMR in the same solution. This increases both sensitivity and accuracy, particularly in the analysis of complex mixtures, and eliminates issues of signal overlap in the densely populated proton spectrum. Moreover, by employing deuterated solvents such as isopropanol‐d_6_, the degree of protonation or deuteration can be determined directly from the NMR spectrum without the need for additional weighing [22] (Figure S2). Recently, this type of heteronuclear calibration using the deuterium solvent signal of D_2_O has been exemplarily applied to plant extracts and pharmaceutical analysis [23, 24].

**FIGURE 2:**
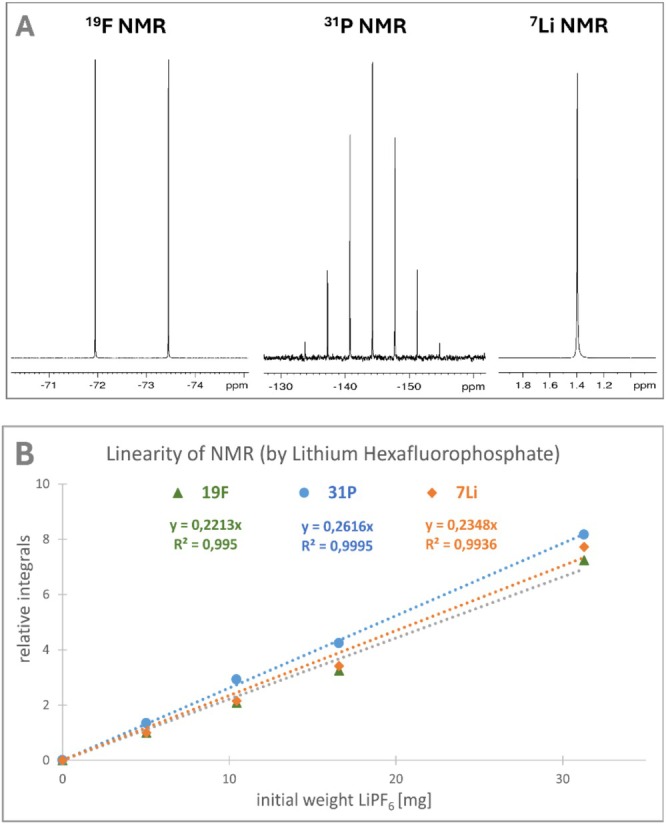
(A) ^19^F‐, ^31^P‐, and ^7^Li‐NMR spectra of lithium hexafluoro phosphate extracted from regular Li‐Ion batteries. (B) ^19^F‐NMR (green), ^31^P‐NMR (blue), and ^7^Li‐NMR (orange) linear regression calibration, relative integral values versus initial weight of a standard lithium hexafluorophosphate [mg] in dilution series.

Particularly, qNMR plays a crucial role in the quantitative analysis of lithium‐ion batteries, especially for studying ion dynamics, electrolyte stability, and solid‐state electrolytes. A key application of our HERMETRIC principle is the analysis of lithium hexafluorophosphate (LiPF₆), the core component of these batteries, providing essential insights into its stability, decomposition pathways, and overall impact on battery performance. In this context, all three active nuclei—^19^F, ^31^P, and ^7^Li—can be employed for quantitative analysis. In particular, the quantification of lithium, a light element, offers a niche for NMR techniques. As expected, all three nuclei show linear correlations with the total amount of the complex salt, following the linear equation y = mx. Due to selective chemical shifts, degradation products such as di‐ and monofluorophosphate can be determined without prior separation, and even the proportion of polyvinyl fluoride can be quantified in acetone‐d₆ (Figure 2).

### 2.4 qNMR Compared With Redox Titration

Titration is an essential part of conventional quantitative analysis and relies on calibrated solutions; therefore, it is not considered a primary method. In contrast, qNMR requires no external calibration and offers a universal quantitative determination independent of reactivity or indicators. Although titration is more sensitive, qNMR delivers higher precision and universal applicability. Furthermore, heteronuclear qNMR enables the monitoring of chemical reactions and their kinetics. While classical titrations are often optimized for clearly detectable end points—for example, in manganometry or iodometry—and frequently require back titration, numerous reactants such as permanganate (^55^Mn‐NMR) or iodine (^127^I‐NMR) can be directly detected in the NMR spectrum.

For example, titrations are the most common methods used in lipid analysis to determine fat quality indices. However, all these titrations can now be replaced by superior NMR‐based methods, including the determination of iodine value [25], peroxide value [26], acid value [27], and anisidine value [28], offering greater precision, efficiency, and reproducibility.

**FIGURE 3:**
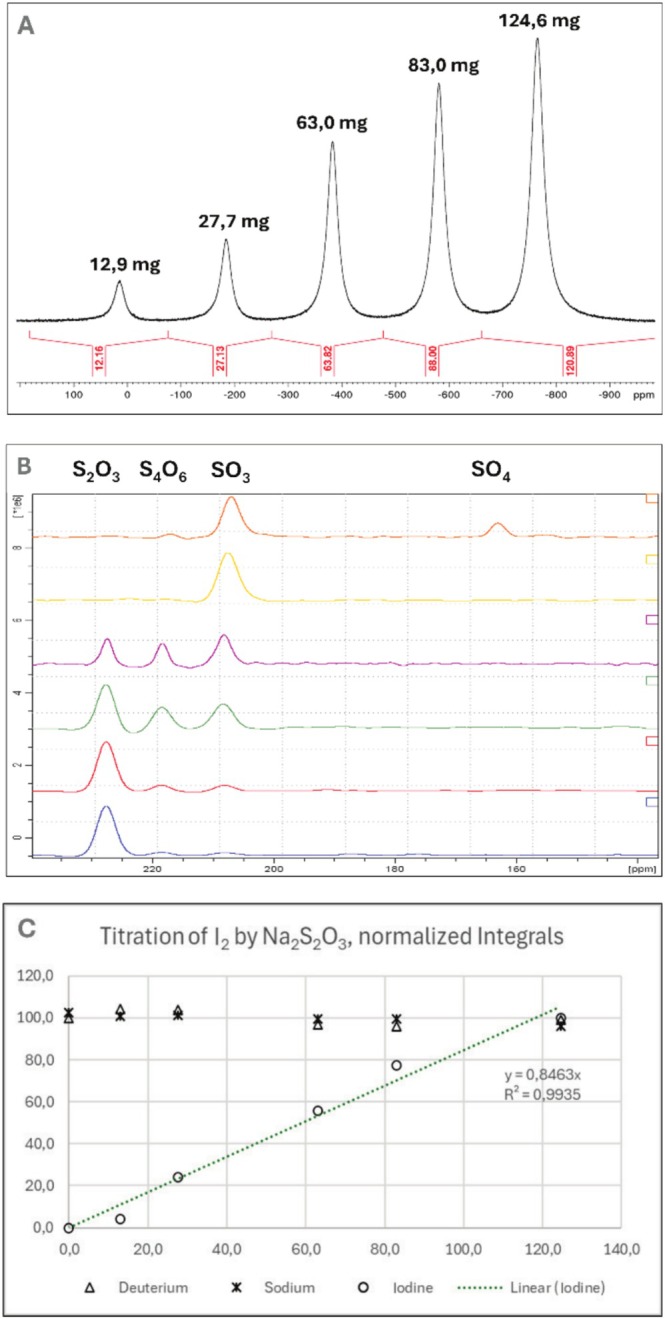
(A) Shifted overlay of ^127^I‐NMR spectra during the reaction of iodine with thiosulfate after addition of x mg I_2_ to 250 mg Na_2_S_2_O_3_*5H_2_O in 1 mL D_2_O. (B) Overlay of ^17^O‐NMR spectra during the reaction of iodine with thiosulfate shows two reaction products, sulfite and dithiosulfate. Excess iodine leads to further oxidation to sulfate. (C) Parallel heteronuclear measurement of ^127^I‐, ^23^Na‐, and ^2^H‐NMR during the reaction of iodine with thiosulfate. The absolute integrals were normalized to 100%. The concentration of sodium and D_2_O is constant. The slight but continuous decrease in these integrals is indicative of volume contraction following the addition of larger amounts of iodine.

A notable example is the reaction of iodine with sodium thiosulfate, where the complex conversion from thiosulfate to dithiosulfate, sulfite, and finally sulfate under an excess of I_2_ is clearly demonstrated. Addition of different amounts of I_2_ to 250 mg Na_2_S_2_O_3_*5H_2_O in 1 mL D_2_O was performed. The chemical reaction can be graphically followed by NMR spectra (Figure 3).

For routine applications, the nuclei ^127^I or ^17^O are less attractive; however, by employing alternative reagents, such titrations can be adapted to an NMR basis—for instance, in the case of the determination of hypochlorite (HClO) where DMSO is used as a substitute for thiosulfate in back titration, enabling the selective and sensitive detection of the resulting dimethyl sulfone (DMS) in the ^1^H‐ and ^17^O‐NMR spectra, while the increasing chloride is monitored by either ^35^Cl or ^37^Cl‐NMR (Figure S3).

This procedure was also applied to manganometry. Due to the symmetric molecular structure of the MnO₄^−^ anion, permanganate exhibits a very intense and narrow signal in ^55^Mn‐NMR, which also enables the detection of the 0.8% ^18^O isotope signal (Figure 8E). The sensitivity is sufficient to directly assess the classical chemical oxygen demand. Furthermore, back titration of low permanganate concentrations can be accelerated by the chemical conversion of DMSO to DMS. In this process, one mole of MnO₄^−^ is effectively transferred into a proton signal, which benefits from a sixfold higher number of active nuclei as well as the significantly greater sensitivity of ^1^H compared with ^55^Mn.

### 2.5 qNMR Compared With Complexometry

**FIGURE 4:**
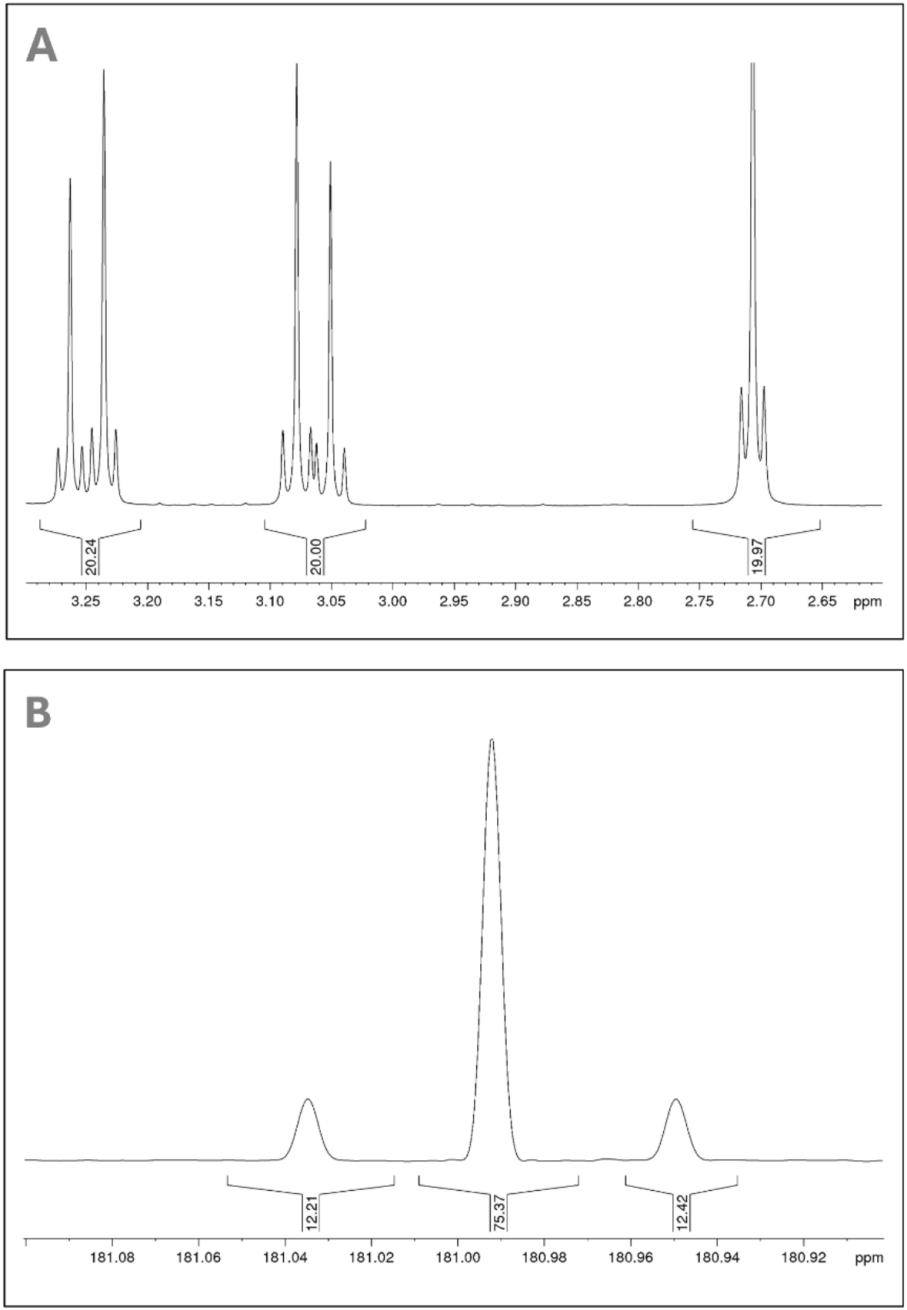
(A) ^1^H‐NMR of a Cd‐EDTA complex in D_2_O, diastereotopic AB system of acetic CH_2_, singlet of ethylenediamine part. Both couplings are caused by spin ½ ^111^Cd and ^113^Cd. (B) ^13^C‐NMR of a Cd‐EDTA complex in D_2_O, carbonyl region. ^111^Cd = 12.5%, ^113^Cd = 12.22%; both spins = ½.

Complexometry, such as the determination of alkaline earth metal salts via EDTA titration, represents another approach to quantitative analysis [29, 30]. Although alkaline earth metals are difficult to access directly via NMR, their behavior in the presence of complexing agents like EDTA can be well quantified. In a basic, aqueous environment, nearly all divalent metal ions form stable, defined EDTA complexes that exhibit characteristic chemical shifts. The integrals of the corresponding proton signals correlate directly with the molar fraction of the metal ion, a phenomenon often enhanced by the molecular geometry. Studies on metal ions such as Sn, Pb, Cd, and Hg confirm the covalent character of these complexes through detectable couplings in the ^1^H‐ and ^13^C‐NMR spectra, as demonstrated, for example, in the cadmium‐EDTA complex (Figure 4). A practical application is the analysis of milk, where the addition of a 20% Cs EDTA solution (pH 9) in D_2_O allows for the determination not only of the fat and lactose content but also of calcium and magnesium, thereby addressing all primary quality criteria (Figure S4).

### 2.6 Volume Contraction and Non‐Standardized NMR Tubes

External calibration methods—such as pulse length–based concentration determination (PULCON) [31] or electronic reference to access in vivo concentrations (ERETIC) [32]—are complicated by the variability in NMR tube volumes as well as the volume contraction of highly concentrated solutions. The latter can be corrected by normalizing the targeted integral by the integral of the deuterium signal, which serves as a function of the concentration in the NMR tube. By dividing the heteronuclear NMR signal (X‐nucleus) by the deuterium signal, the “per liter” factor is eliminated, yielding a volume‐independent quantity. For quantification, one can proceed with the mass of analytes. This significantly simplifies sample preparation—it is sufficient to add a defined volume of deuterated solvent (e.g., exactly 1 mL) without relying on a fixed fill volume. Besides simpler sample preparation, no specific attention should be kept on NMR‐tubes quality while measuring a series of samples.

## 3 Further Investigations on Inorganic Salts

As part of the holistic NMR analysis of heparin, we have determined not only the biopolymer content but also the water content and the concentrations of inorganic counterions (sodium [Na^+^] and chloride [Cl^−^]) using qNMR. Calibration was performed against the solvent signal of D_2_O via ^2^H‐NMR, enabling a precise and traceable quantification. This approach allows simultaneous analysis of multiple parameters without the need for additional chemical modifications or external calibration standards. The holistic methodology serves as an alternative to conventional techniques such as Karl Fischer titration or ion chromatography, offering precise, non‐destructive, and SI‐traceable quantification, with all analyses obtained from a single sample preparation within one NMR tube [33].

In organic chemistry, the nuclear ratios within a compound are fixed, which enables consistent calibration of all active NMR nuclei. Our aim is to extend this approach to the entire periodic table in order to quantitatively analyze inorganic components by NMR. To this end, we have replaced suitable molecules with salts that have a defined molar composition. While alkali and halogen metals can be directly analyzed by NMR, other elements are less suitable due to their low natural abundance or unfavorable magnetic properties.

Our investigations were initially focused on sodium chloride nuclei, whose ratio in solution always remains 1:1. From any sodium chloride solution, the response in ^23^Na‐ and ^35^Cl‐NMR between the two nuclei can be determined—taking into account fundamental physicochemical constants such as natural abundance, relative sensitivity, gyromagnetic ratio, quadrupolar behavior, and experimental parameters such as number of scans (NS), shimming, tuning and matching, and receiver gain (RG). The latter should be kept constant, where possible, for a given NMR spectrometer. From the absolute signal intensity (considering NS and RG), a virtual single‐pulse signal for each nucleus can be defined. An extension to potassium chloride additionally enables the determination of the response among sodium, potassium, and chloride. Particularly advantageous is the sensitive cesium NMR signal, which exhibits a 200‐fold higher sensitivity than carbon and serves as an internal standard for alkaline earth determinations. Through a second cascade, all sodium–halide salts can be analyzed—for instance, a 1 M solution of sodium chloride and potassium bromide yields the same qNMR spectrum as a 1 M solution of sodium bromide and potassium chloride, as confirmed experimentally by measurements of ^23^Na, ^39^K, ^35^Cl, ^37^Cl, ^79^Br, and ^81^Br (Figure S5).

Expanding this concept to include a direct response factor *rfₓ* between the observed NMR nucleus and the deuterium nucleus of the solvent, the ratio of the respective virtual single‐pulse integrals *Int*(*X*) can be expressed, taking into account the present molar quantity *n* and the defined amount of deuterium nuclei in exactly 1 mL of D_2_O (equivalent to 110.5 mmol of ^2^H), according to Equation (1):

(1) $$\frac{\mathrm{Int}\left(X\right)}{n*N_{x}}*{\mathrm{rf}}_{X}=\frac{\mathrm{Int}\left(D\right)}{0.1105}$$

This equation simplifies the determination of the response factor for ^23^Na, ^39^K, ^35^Cl, ^37^Cl, ^79^Br, and ^81^Br with respect to deuterium (*D*) in the case of 1 M salt solutions, provided that complete solubility of the salt and correction for volumetric contraction are ensured. On the same basis, given a known response factor, the molar amount of monovalent salts in aqueous solution in exactly 1 mL of D_2_O is determined by:

(2) $$n_{x}=\frac{{\mathrm{rf}}_{x}*\mathrm{Int}\left(x\right)*0.1105}{\;\mathrm{Int}\left(D\right)}$$

For practical application, eight salt solutions were prepared and direct NMR measurements of the six mentioned nuclei, as well as ^1^H and *D*, were performed on the same day, using the same instrument and identical measurement parameters, within a short time span (Table S1).

**FIGURE 5:**
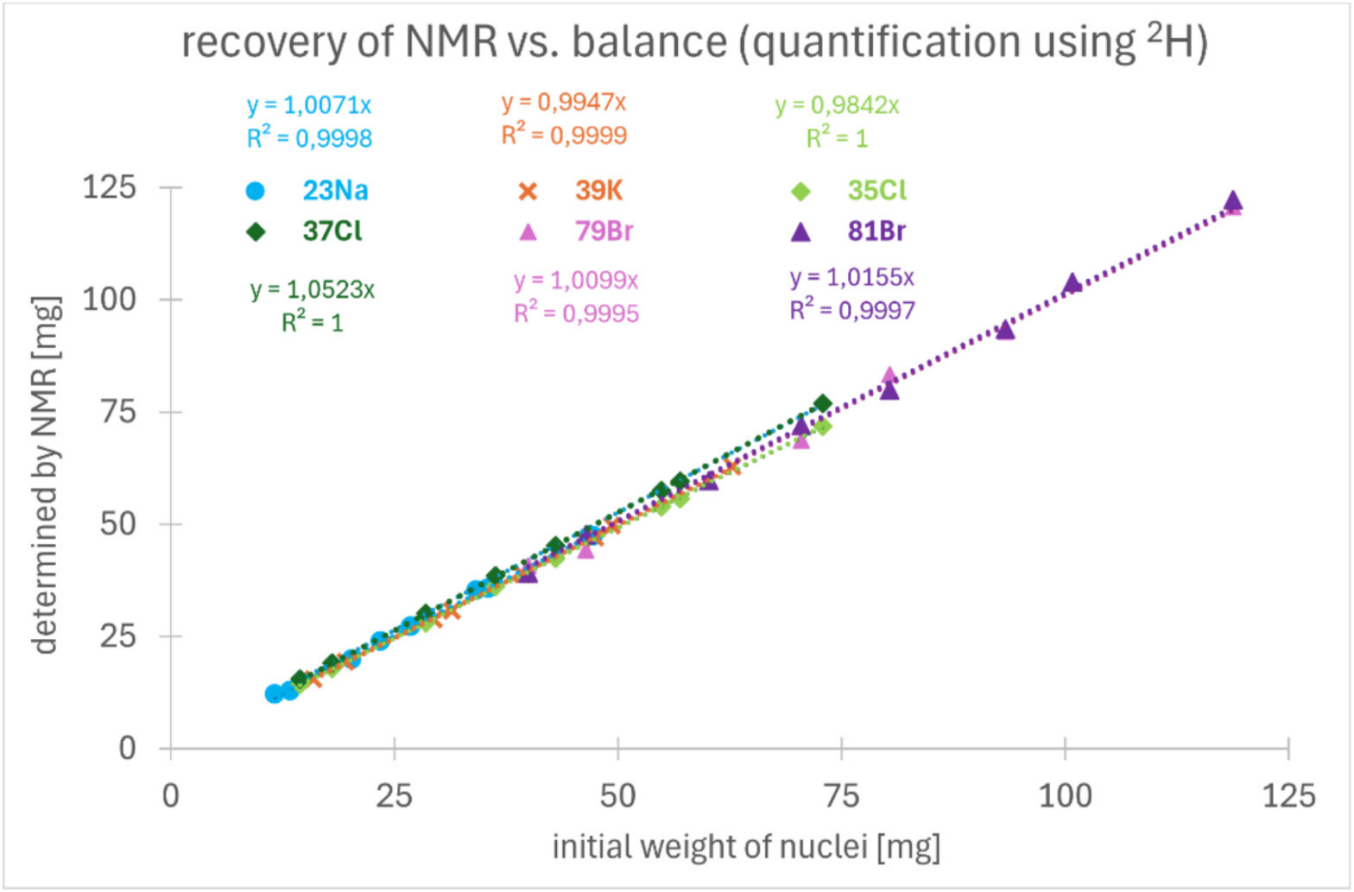
Linear regression (recovery) of NMR determined amount [mg] versus initial weight [mg] of investigated nuclei using ^23^Na‐, ^37^Cl‐NMR (left), ^39^K‐, ^79^Br‐NMR (middle), and ^35^Cl‐, ^81^Br‐NMR (right) of model salt solution in 1 mL D_2_O.

Using Equation (2), the molar quantity of the precisely stoichiometrically defined salts could be determined solely based on the absolute integrals from the NMR measurements and the respective response factors. The NMR‐determined quantities of Na, Cl, K, and Br, calculated via molecular weight, were plotted against the actual weighed‐in masses (Figure 5). The clearly linear correlations were observed for all six investigated nuclei, demonstrating that this approach of internal calibration can be successfully applied in principle and easily extended to other NMR‐active nuclei.

## 4 ^17^O‐NMR [34]: A Variant That Is Only Seemingly Exotic

**FIGURE 6:**
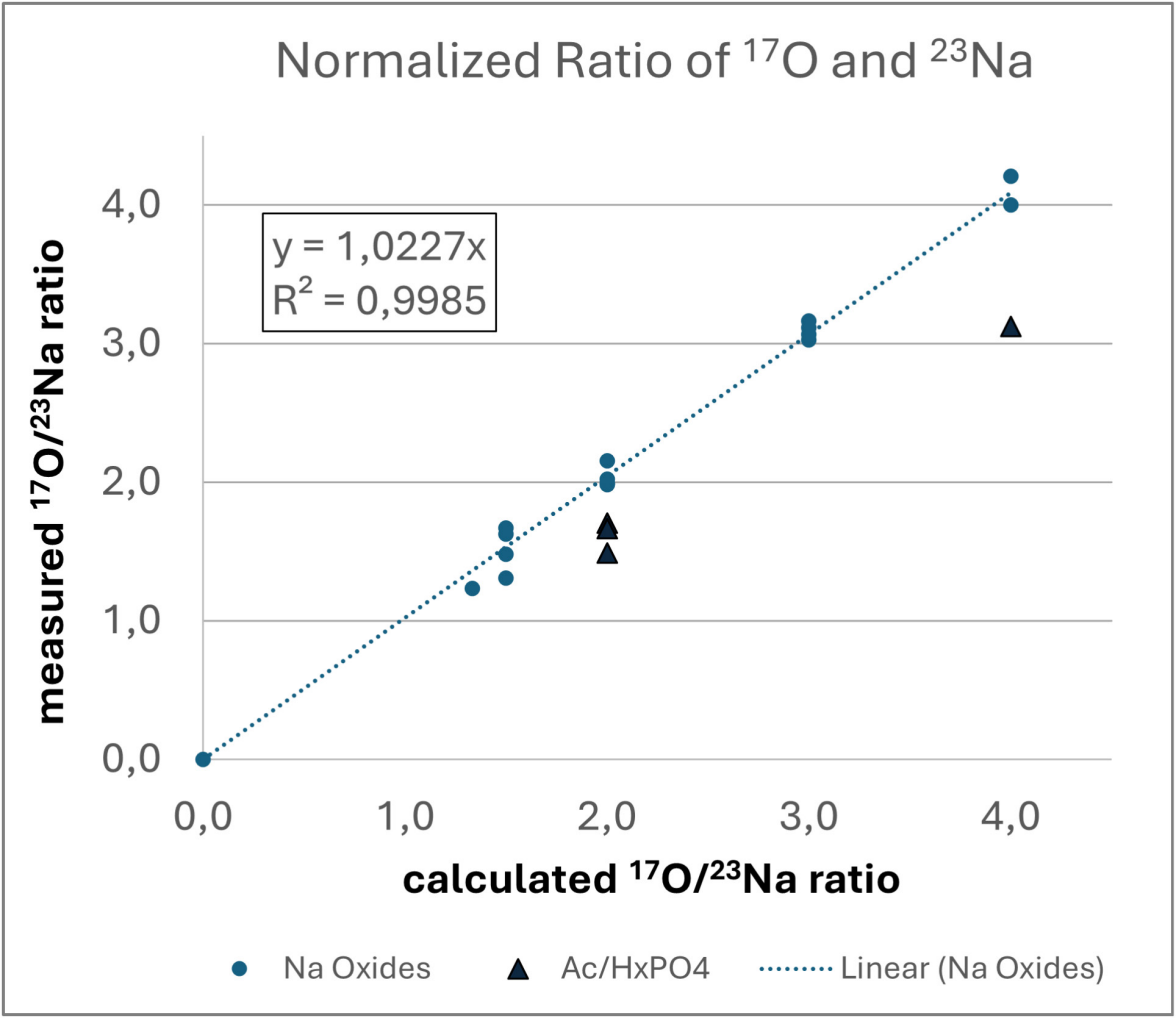
Linear correlation of calculated and analyzed ratios of ^23^Na‐ and ^17^O‐NMR signals of several oxides. The measured ratios for acetates and hydrogen phosphates deviate slightly and are marked as triangles. These four results are excluded from linear regression.

Another focal point is the study of more complex inorganic compounds, in which isotope ^17^O plays a central role. Only a few qNMR studies focus on the ^17^O nucleus in organic material [35, 36, 37]. Although ^17^O‐NMR is not a routine application due to its low natural abundance, it provides deeper insights into analytical processes such as redox titrations. Various sodium salts of oxygen‐containing anions have been investigated, and the stoichiometric relationships between the ^23^Na‐ and ^17^O‐NMR spectra exhibited a linear correlation under identical conditions. Deviations observed with weak bases such as carbonates or with imprecisely defined hydrogen phosphates can be chemically explained (Figure 6, Table 1, Figure S6).

**TABLE 1:** Calculated and analyzed ratio of ^23^Na‐ and ^17^O‐NMR signals of several oxides. The measured ratios for acetates and hydrogen phosphates deviate slightly, likely due to deviations from stoichiometry.

Substance	Calculated	Measured
D_2_O	0.0	0.0
NaMnO_4_	4.0	4.0
Na_2_CO_3_	1.5	1.3
Na_2_S_2_O_3_*5H_2_O	1.5	1.5
Na_2_CrO_4_	2.0	2.0
Na_3_PO_4_*5H_2_O	1.3	1.2
Na_2_SO_3_	1.5	1.6
NaClO_3_	3.0	3.1
NaH_2_PO_2_	2.0	2.0
NaNO_3_	3.0	3.1
NaNO_2_	2.0	2.0
Na_2_S_2_O_8_	3.0	3.0
Na_2_SO_4_	2.0	2.0
Na_2_FPO_3_	1.5	1.7
Na_2_MoO_4_*2H_2_O	2.0	2.2
Na‐isethionate	3.0	3.2
NaClO_4_*H_2_O	4.0	4.2
NaAc*3H_2_O	2.0	1.7
NaAc	2.0	17
NaH_2_PO_4_	4.0	3.1
Na_2_HPO_4_	2.0	1.5

Potential applications of these investigations primarily arise in the monitoring of redox processes as shown above as well as in Karl Fischer water determination. The theoretically prescribed linear relationships between ^23^Na‐, ^55^Mn‐, and ^17^O‐NMR within a dilution series were experimentally confirmed, and reduction reactions—such as the reduction of sodium permanganate, iodine, or bromine—were monitored using ^127^I‐, ^23^Na‐, and ^17^O‐NMR.

**FIGURE 7:**
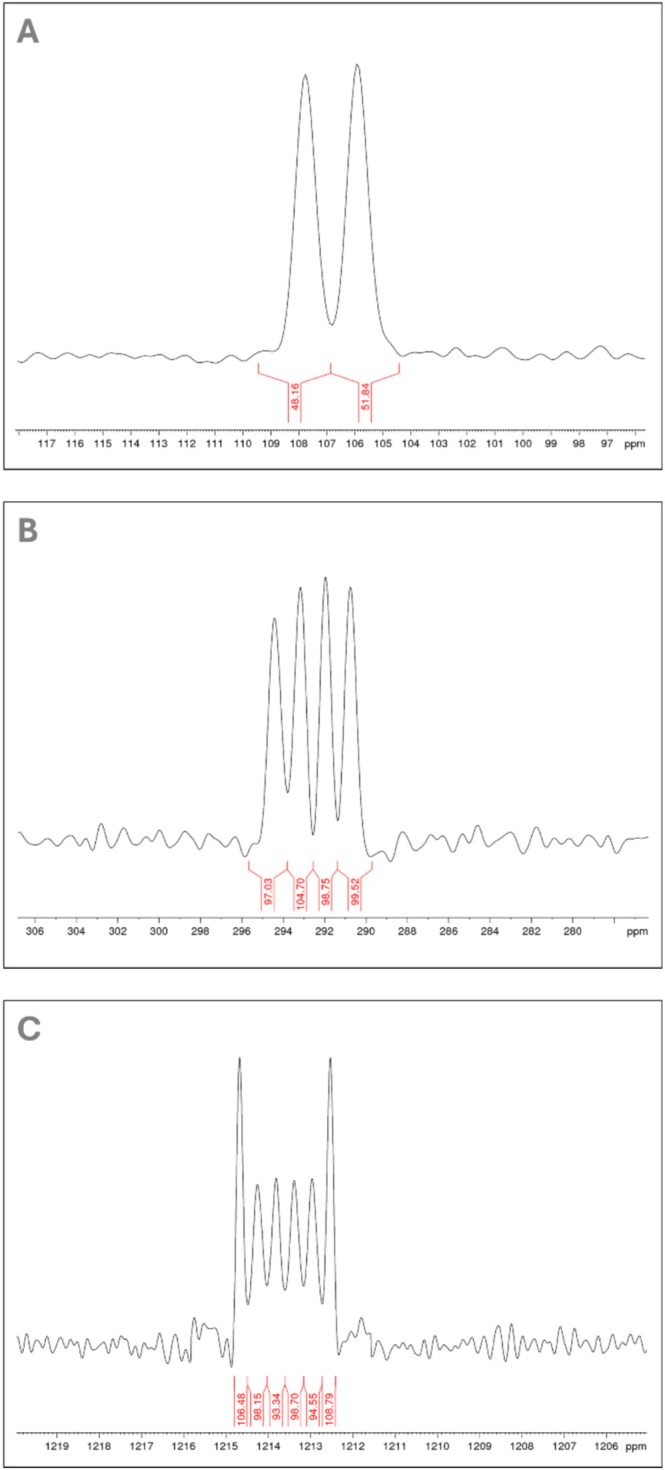
(A) High resolution ^17^O‐NMR spectrum of PO_4_, 200 mg/mL D_2_O, a classical doublet by coupling with spin = ½. (B) High resolution ^17^O‐NMR spectrum of ClO_4_, 200 mg/mL D_2_O, quadrupole coupling with spin 3/2 for ^35^Cl and also ^37^Cl shows four signals with an integral ratio of 1:1:1:1. Different intensities of this quartet are caused by the quadrupole orientation in the magnetic field. (C) High resolution ^17^O‐NMR spectrum of MnO_4_, 200 mg/mL D_2_O, quadrupole coupling with spin 5/2 shows six signals with an integral ratio of 1:1:1:1:1:1. Different intensities of sextet are caused by the quadrupole orientation in the magnetic field.

It is necessary to further emphasize the ^17^O‐NMR spectra, which possess considerable aesthetic value and were described in earlier publications [38]. Although challenging to acquire, these spectra—when obtained under optimal acquisition conditions and properly processed—reveal signals complete with the corresponding coupling patterns, especially with quadrupole nuclei. These quantum‐mechanically based effects attest to a comprehensive and holistic understanding of NMR spectroscopy, and they are unequivocally quantitative [39] (Figure 7).

## 5 Isotope Effects From Active and Inactive NMR Nuclei in Covalent Compounds

In mass spectrometry (MS), signals are detected beyond the nominal mass that are caused by the presence of different isotopes of the same element. These signals serve as indicators for the presence of specific atoms in organic molecules. For example, the characteristic patterns produced by ^35^Cl and ^37^Cl or by ^79^Br and ^81^Br in halogens are well known and are already part of the basic training in MS interpretation. Using NMR spectroscopy, it is also possible to observe similar effects on signal splitting of well‐observable active nuclei. For example, most NMR spectroscopists have already noticed the splitting of the ^19^F‐NMR signal of the commonly used reference standard Cl_3_CF due to the isotopic distribution of ^35^Cl and ^37^Cl, analogous to what is observed in MS. This phenomenon can be further observed for ^19^F and ^13^C chemical shifts in bromine‐substituted organic molecules and represents a general effect of heavier isotopes influencing lighter ones, a well‐known manifestation of isotopic effects in spectroscopy [40, 41, 42] (Figure S7).

Using this approach, other elements can be qualitatively determined. Although all halogens—and even all their various isotopes—are NMR‐active, only ^19^F atoms are readily detectable in organic and inorganic molecules due to their spin‐½ nature. The other halogens, being quadrupolar nuclei, are observable only in highly symmetric anions, such as X^−^ or XO₄^−^ [43]. Nevertheless, chlorine and bromine leave an isotopic pattern in organic molecules because the heavier isotopes shift the chemical shifts of their covalently bound atoms toward higher field. In organic chlorides or bromides, this can be clearly observed in high‐resolution ^13^C‐ or ^19^F‐NMR spectra.

**FIGURE 8:**
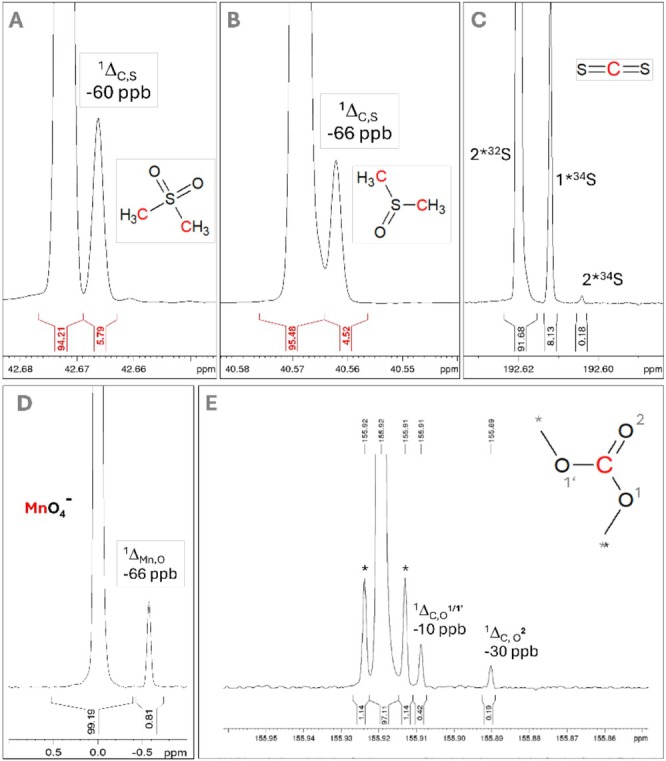
(A) ^13^C‐NMR excerpt of DMS, (B) ^13^C‐NMR excerpt of DMSO, (C) ^13^C‐NMR excerpt of CS_2_. The number of sulfur can be calculated by integration of the ^34^S isotope signals. Comparing the DMSO and DMS indicates that the amount of ^34^S is not equivalent. (D) ^55^Mn‐NMR of NaMnO_4_, (E) ^13^C‐NMR of dimethyl carbonate. The number of oxygens can be calculated by integration of the ^18^O isotope signals.

In the case of other elements, which isotopes have low natural abundance, such as ^17^O, ^33^S, ^15^N, or ^29^Si—which occur in many organic compounds—or even in some more exotic tin organometallics (where ^117^Sn, ^119^Sn, and ^121^Sn each have a nuclear spin of ½), the isotopic ratio can be quantitatively determined by analyzing ^13^C‐NMR spectra. Leaving aside the more exotic compounds, the two nuclei most important in chemistry remain oxygen and sulfur. Although both possess an NMR‐active nucleus, their concentrations are very low. As shown above, ^17^O is suitable for direct observation in organic and inorganic molecules and salts, albeit only to a limited extent. But what about the widespread isotopes with nuclear spin *n* = 0—such as ^28^Si and ^30^Si, ^16^O and ^18^O, ^32^S and ^34^S? In the case of ^12^C, it is generally assumed that this isotope is not directly visible in the NMR spectrum. But is that really so? The mass spectrometrist is well aware that the sulfur content in a compound can be determined from the M + 2 signal—as a rule of thumb, every 5% M + 2 corresponds to one sulfur atom in the molecule. The proportion of ^13^C is estimated via the M + 1 peak, and the silicon pattern is also characteristic, even when high‐resolution MS is not employed (Figure 8). Carbon atoms exhibit two distinct signals in a ratio of approximately 95.0–4.2 when bound to different isotopes of sulfur. The sensitivity of the NMR measurement is sufficient to detect deviations from the statistically expected proportion of ^34^S.

These observations can be transferred to other NMR experiments. ^18^O reliably displays a high‐field signal corresponding to 0.2% integral per oxygen in both organic molecules and in phosphates (^31^P‐NMR), permanganates (^55^Mn‐NMR), or perchlorates (^35^Cl or ^37^Cl‐NMR)—even within the ^13^C‐NMR signal of CO_2_ dissolved in CDCl_3_. Admittedly, these observed effects have little practical application so far, but they nevertheless demonstrate the absolutely quantitative, ab initio capabilities of NMR spectroscopy—provided that the experimental conditions and the signal‐to‐noise ratio are appropriately chosen. Site‐specific natural isotope fractionation nuclear magnetic resonance (SNIF‐NMR) is a highly precise technique for determining isotopic distributions in organic molecules [44]. These isotopic signatures, shaped by biosynthesis and environmental factors, help verify authenticity, origin, and production methods [45].

SNIF‐NMR measures isotope ratios at specific molecular positions, with ^2^H‐NMR analyzing deuterium distribution and ^13^C‐NMR providing complementary carbon isotope data. This method is already applied to substances like vanillin [46], sucrose [47], or nicotine [48] with ongoing research in forensic detection of counterfeit pharmaceuticals. This methodology could also be extended to other nuclei such as oxygen, sulfur, or nitrogen if the corresponding isotope fractionations exhibit significant differences between natural and synthetic sources and a reliable NMR detection of these isotopes is feasible.

## 6 Outlook on Future Developments and Harmonization of qNMR

From a metrological perspective, it is not practical to replace all established analytical methods with NMR; however, our considerations and experiments underscore the fundamental properties of qNMR. Through a cascade of response measurements on stoichiometrically defined molecules and salts, it is possible to trace all nuclei back to deuterium as the primary standard defined by 110.5 mmol of deuterium in 1 mL of D_2_O. Although the NMR periodic table of elements may not fully capture the qNMR periodic table in every case, the principles of this primary method should foster a new understanding in analytical chemistry. All applications discussed in this contribution require modern high‐resolution NMR equipment, which is tuned to different nuclei and sensitive enough to observe fine isotopic patterns. Consequently, in the future, more qNMR methods could be applied as alternatives or supplements to classical analytical techniques—such as in pharmacopoeias and other standard works.

## 7 Experimental

All NMR measurements on heteronuclei were performed on a Bruker 500 MHz RT instrument with a BBFO probe. The high‐resolution ^13^C‐NMR spectra were recorded on a Bruker 600 MHz TCI cryo instrument. The measurement parameters must be individually adjusted to the properties of the instruments and the isotopes under investigation. The data that support the findings of this study are available from the corresponding author upon reasonable request.

## Supporting information


**Figure S1:** (A) ^1^H‐NMR spectrum of TCNB (int. Std.) and TPP at 600 MHz. (B) Statistical data evaluation of laboratory staff.
**Figure S2:** (A) 1H‐NMR. (B) 2H‐NMR, single pulse spectra at 500 MHz of 100 μL isopropanol‐d_6_ in 1 mL CDCl_3_ to calibrate the degree of deuteration.
**Figure S3:** (A) ^17^O‐NMR during the reaction of hypochlorite (ClO‐) with DMSO to chloride and DMS. ClO‐ is not detectable, but chlorate (ClO_3_‐) as degradation product of ClO‐ is in ^17^O‐NMR. (B) Increasing amount of chloride during the reaction of hypochlorite with DMSO to DMS. Hypochlorite solutions show large initial amounts of chlorate (shown in ^17^O‐NMR) and chloride by ^35^Cl‐NMR. (C) Increasing amount of DMS during the reaction of hypochlorite with DMSO by ^1^H‐NMR.
**Figure S4:** (A) Quantitative analysis of calcium and magnesium in whole milk, 800 μL milk, and 200 μL EDTA solution in D_2_O. The pH was adjusted to 9.0 using cesium carbonate. (B) ^1^H‐NMR spectrum, simultaneous analysis of zinc, calcium, and magnesium in a solution of defibrotide (depolymerized porcine intestinal DNA) in D_2_O.
**Figure S5:** Shifted overlays of ^23^Na‐, ^39^K‐NMR (left), ^35^Cl‐, ^37^Cl‐NMR (middle), and ^79^Br‐, ^81^Br‐NMR (right) of 1 M solutions of NaCl + KBr (blue) and NaBr + KCl (green) in D_2_O, with equimolar ratio displayed by integral ratio.
**Figure S6:** mrc70020‐sup‐0001‐Supp_Info.docx. ^17^O‐NMR spectrum of an equimolar mixture from NaNO_3_ (δ 415 ppm) and Na_2_CO_3_ (δ 193 ppm) in D_2_O (δ 0 ppm).
**Figure S7:** mrc70020‐sup‐0001‐Supp_Info.docx. ^19^F‐NMR of Cl_3_CF (left), ^19^F‐NMR of Br_3_CF (middle), and ^13^C‐NMR of Br‐Phenyl ipso C atom (right). The isotope patterns are quantitatively equivalent to mass spectrometry (MS).
**Table S1:** Model mixtures of salts in aqueous solution.

## Data Availability

The data that support the findings of this study are available from the corresponding author upon reasonable request.
