# Supplementary material for: HEteronuclear Referencing for METRologic Isotope Calibration (HERMETRIC)
Source: Magn Reson Chem. 2025 Aug 18;63(11):885–94. doi: 10.1002/mrc.70020 (PMC12500356; doi:10.1002/mrc.70020)
Supplement: Supplementary file 1 — Figure S1: (A) 1H‐NMR spectrum of TCNB (int. Std.) and TPP at 600 MHz. (B) Statistical data evaluation of laboratory staff. Figure S2: (A) 1H‐NMR. (B) 2H‐NMR, single pulse spectra at 500 MHz of 100 μL isopropanol‐d6 in 1 mL CDCl3 to calibrate the degree of deuteration. Figure S3: (A) 17O‐NMR during the reaction of hypochlorite (ClO‐) with DMSO to chloride and DMS. ClO‐ is not detectable, but chlorate (ClO3‐) as degradation product of ClO‐ is in 17O‐NMR. (B) Increasing amount of chloride during the reaction of hypochlorite with DMSO to DMS. Hypochlorite solutions show large initial amounts of chlorate (shown in 17O‐NMR) and chloride by 35Cl‐NMR. (C) Increasing amount of DMS during the reaction of hypochlorite with DMSO by 1H‐NMR. Figure S4: (A) Quantitative analysis of calcium and magnesium in whole milk, 800 μL milk, and 200 μL EDTA solution in D2O. The pH was adjusted to 9.0 using cesium carbonate. (B) 1H‐NMR spectrum, simultaneous analysis of zinc, calcium, and magnesium in a solution of defibrotide (depolymerized porcine intestinal DNA) in D2O. Figure S5: Shifted overlays of 23Na‐, 39K‐NMR (left), 35Cl‐, 37Cl‐NMR (middle), and 79Br‐, 81Br‐NMR (right) of 1 M solutions of NaCl + KBr (blue) and NaBr + KCl (green) in D2O, with equimolar ratio displayed by integral ratio. Figure S6: mrc70020‐sup‐0001‐Supp_Info.docx. 17O‐NMR spectrum of an equimolar mixture from NaNO3 (δ 415 ppm) and Na2CO3 (δ 193 ppm) in D2O (δ 0 ppm). Figure S7: mrc70020‐sup‐0001‐Supp_Info.docx. 19F‐NMR of Cl3CF (left), 19F‐NMR of Br3CF (middle), and 13C‐NMR of Br‐Phenyl ipso C atom (right). The isotope patterns are quantitatively equivalent to mass spectrometry (MS). Table S1: Model mixtures of salts in aqueous solution. [file MRC-63-885-s001.docx]

**Supporting Information:**

**HE**teronuclear **R**eferencing for **METR**ologic **I**sotope **C**alibration **(HERMETRIC)**

Bernd W.K. Diehl*^1,2^, Jakob Waldthausen^1,3^, Yulia Monakhova^1,3,4^

^1^ Spectral Service AG, Emil-Hoffmann-Straße 33, 50996 Cologne, Germany

^2^ University of Applied Sciences Bonn-Rhein-Sieg, Faculty of Applied Sciences, Von-Liebig-Straße 20, 53359 Rheinbach, Germany

^3^ University of Applied Sciences Aachen, Department of Chemistry and Biotechnology, Heinrich-Mußmann-Straße 1, D-52428 Jülich, Germany

^4^ Saratov State University, Institute of Chemistry, Astrakhanskaya Street 83, 410012 Saratov, Russia

* Corresponding author at Spectral Service AG. E-mail address: [bernd.diehl@spectralservice.de](mailto:bernd.diehl@spectralservice.de)


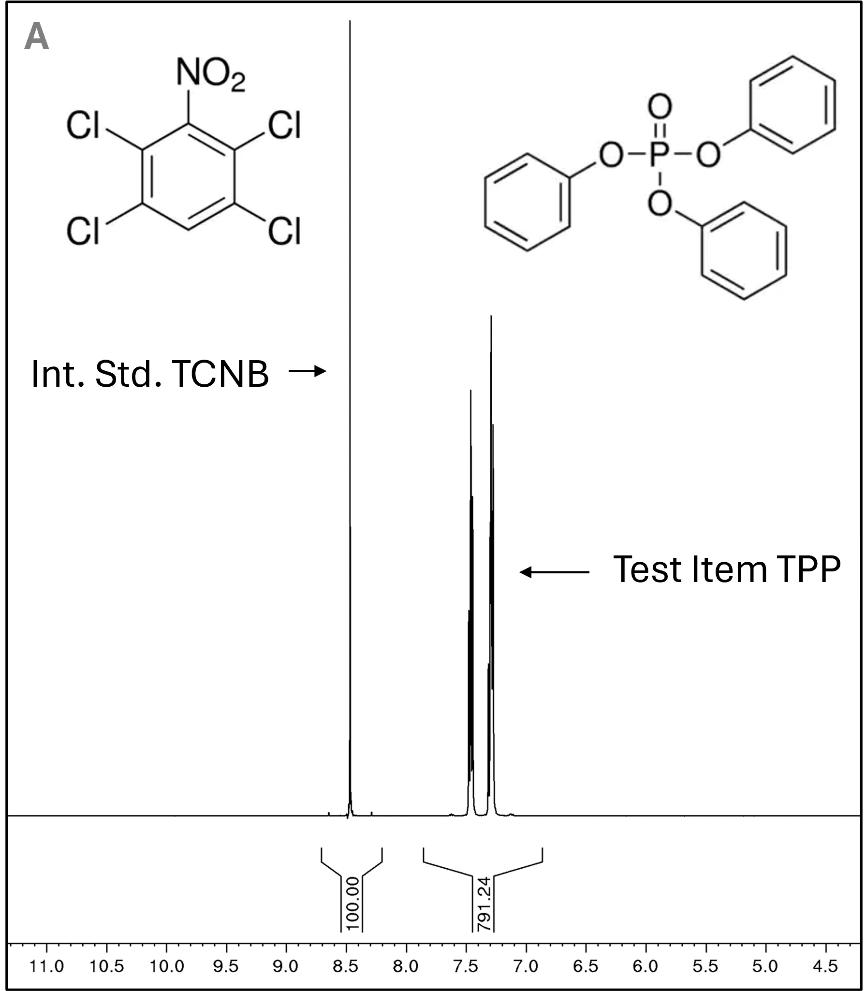


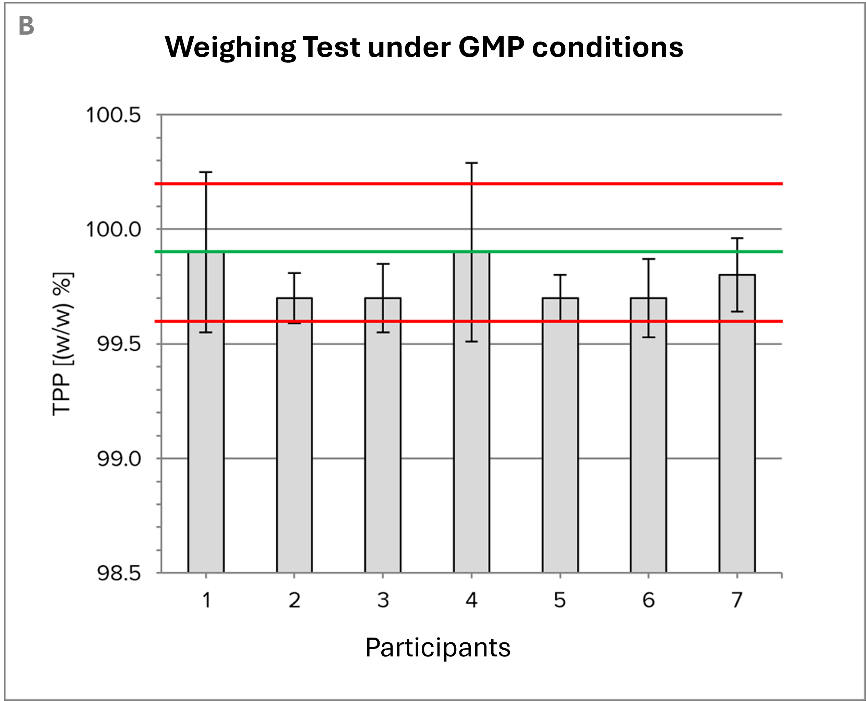


**Fig. S1 (A)** ^1^H-NMR Spectrum of TCNB (int. Std.) and TPP at 600 MHz **(B)** Statistical data evaluation of laboratory staff.


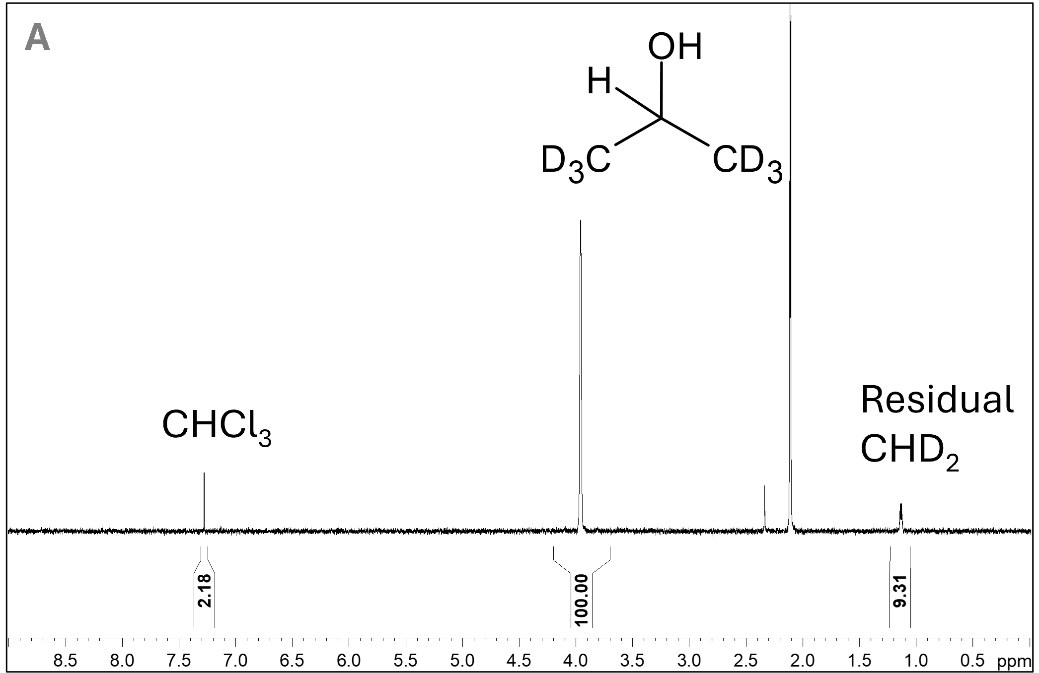


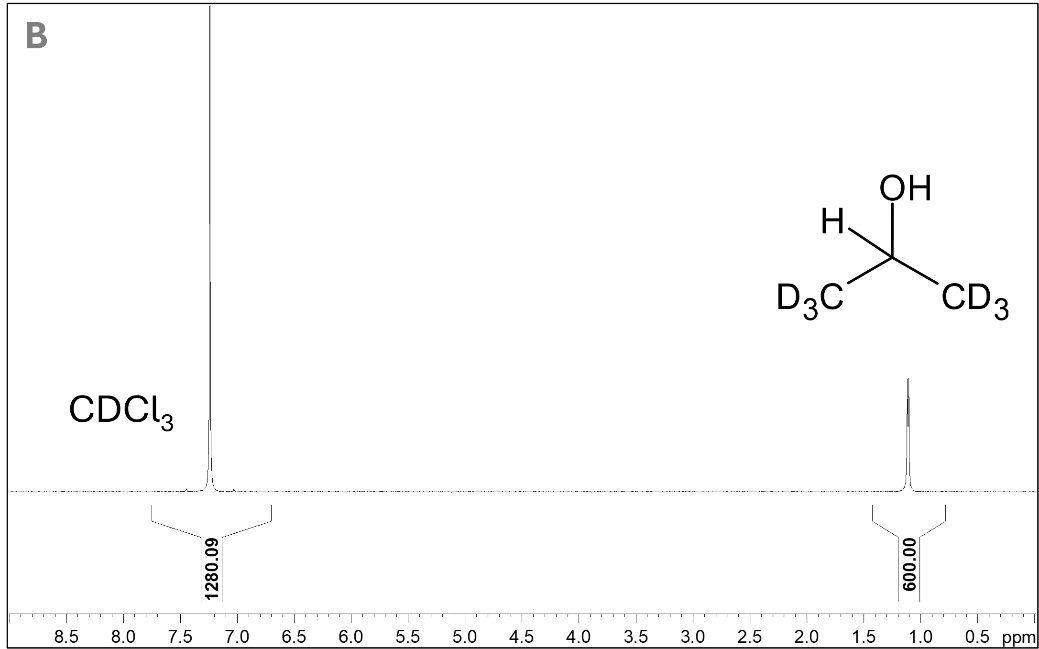


***Fig. S2 (A)*** ^1^H-NMR ***(B)*** ^2^H-NMR, Single pulse spectra at 500 MHz of 100 µl iso-Propanol-d₆ in 1 ml CDCl₃ to calibrate the degree of deuteration.

**
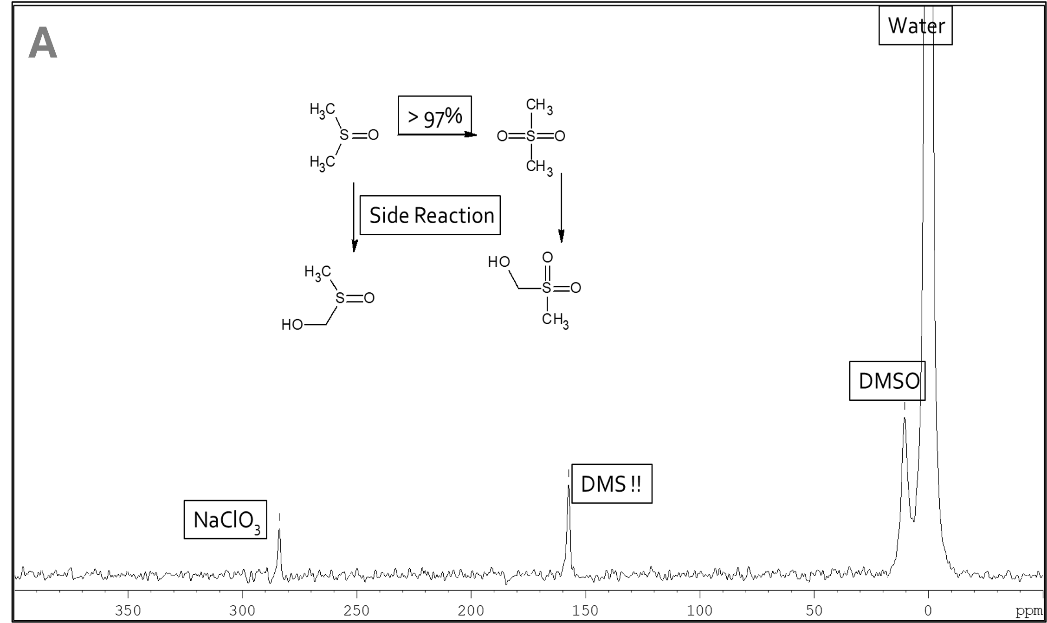
**

**
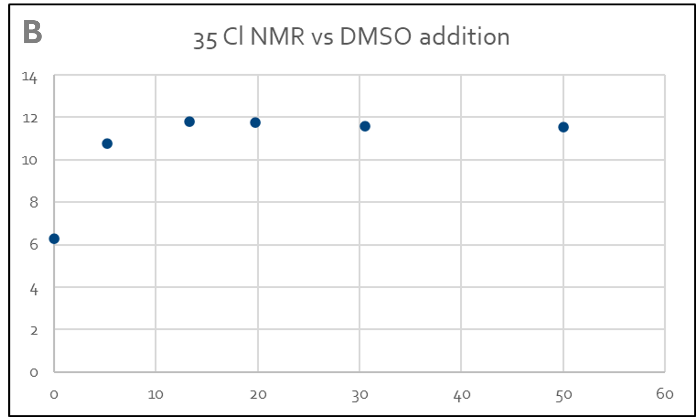
**

**
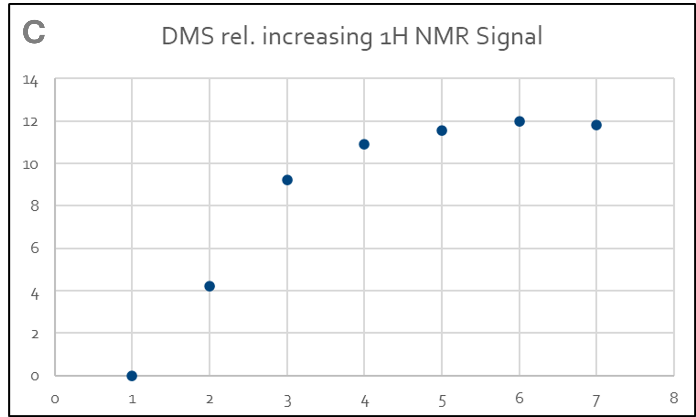
**

***Fig. S3 (A)*** ^17^O-NMR during the reaction of Hypochlorite (ClO^-^) with DMSO to Chloride and DMS. ClO^-^ is not detectable, but Chlorate (ClO_3_^-^) as degradation product of ClO^-^ is in ^17^O-NMR ***(B)*** Increasing amount of Chloride during the reaction of Hypochlorite with DMSO to DMS. Hypochlorite solutions show large initial amounts of Chlorate (shown in ^17^O-NMR) and Chloride by ^35^Cl-NMR. ***(C)*** Increasing amount of DMS during the reaction of Hypochlorite with DMSO by ^1^H-NMR.*
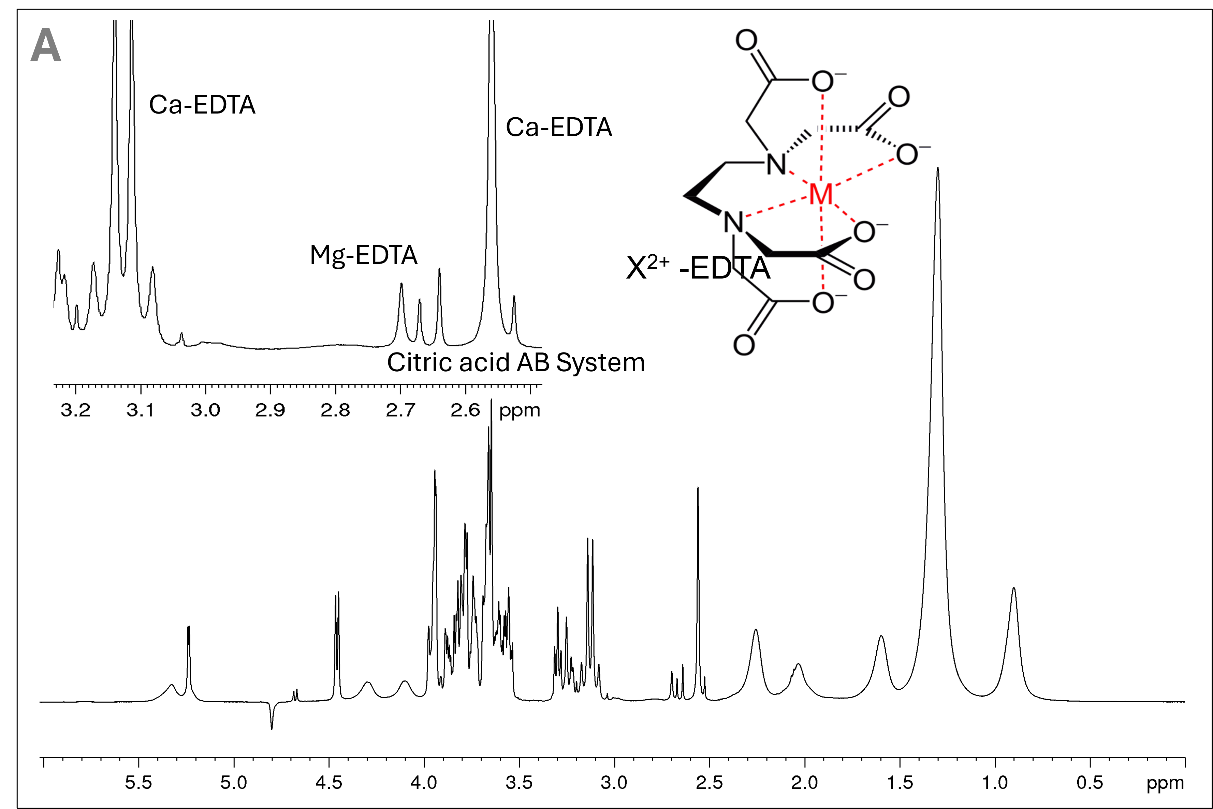
*

***
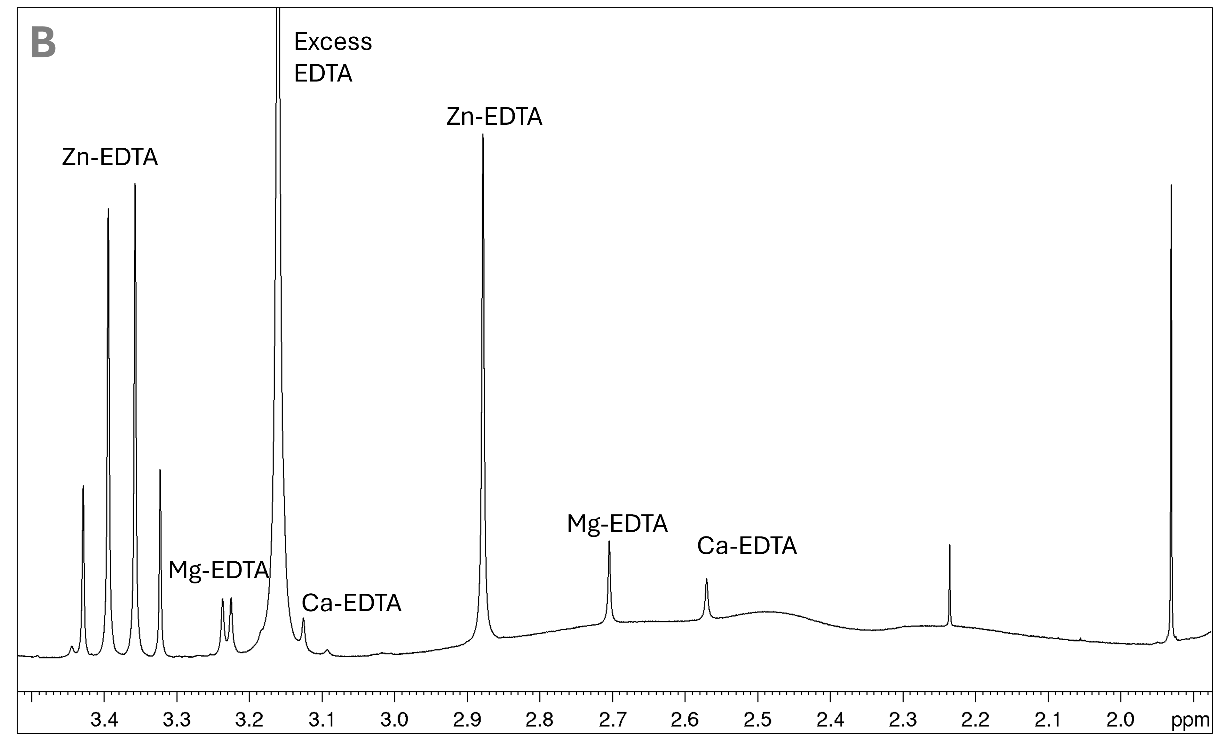
***

***Fig. S4 (A)*** Quantitative analysis of Calcium and Magnesium in whole milk, 800µl milk and 200 µl EDTA solution in D_2_O The pH was adjusted to 9.0 using cesium carbonate. ***(B)*** ^1^H-NMR spectrum, simultaneous analysis of Zink, Calcium and Magnesium in a solution of Defibrotide (depolymerized porcine intestinal DNA) in D_2_O.


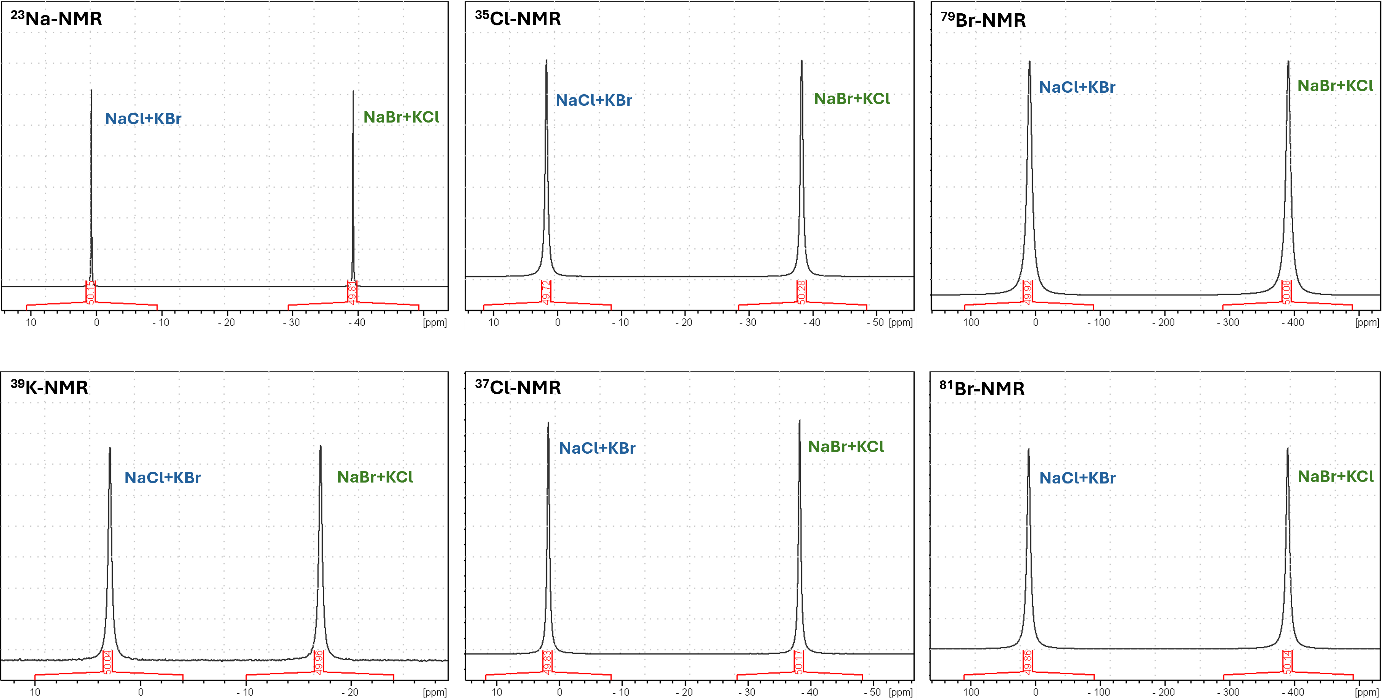


***Fig. S5.*** Shifted overlays of ^23^Na-, ^39^K-NMR (left), ^35^Cl-, ^37^Cl-NMR (middle) and ^79^Br-, ^81^Br-NMR (right) of 1M solutions of NaCl+KBr (blue) and NaBr+KCl (green) in D_2_O, with equimolar ratio displayed by integral ratio.

***
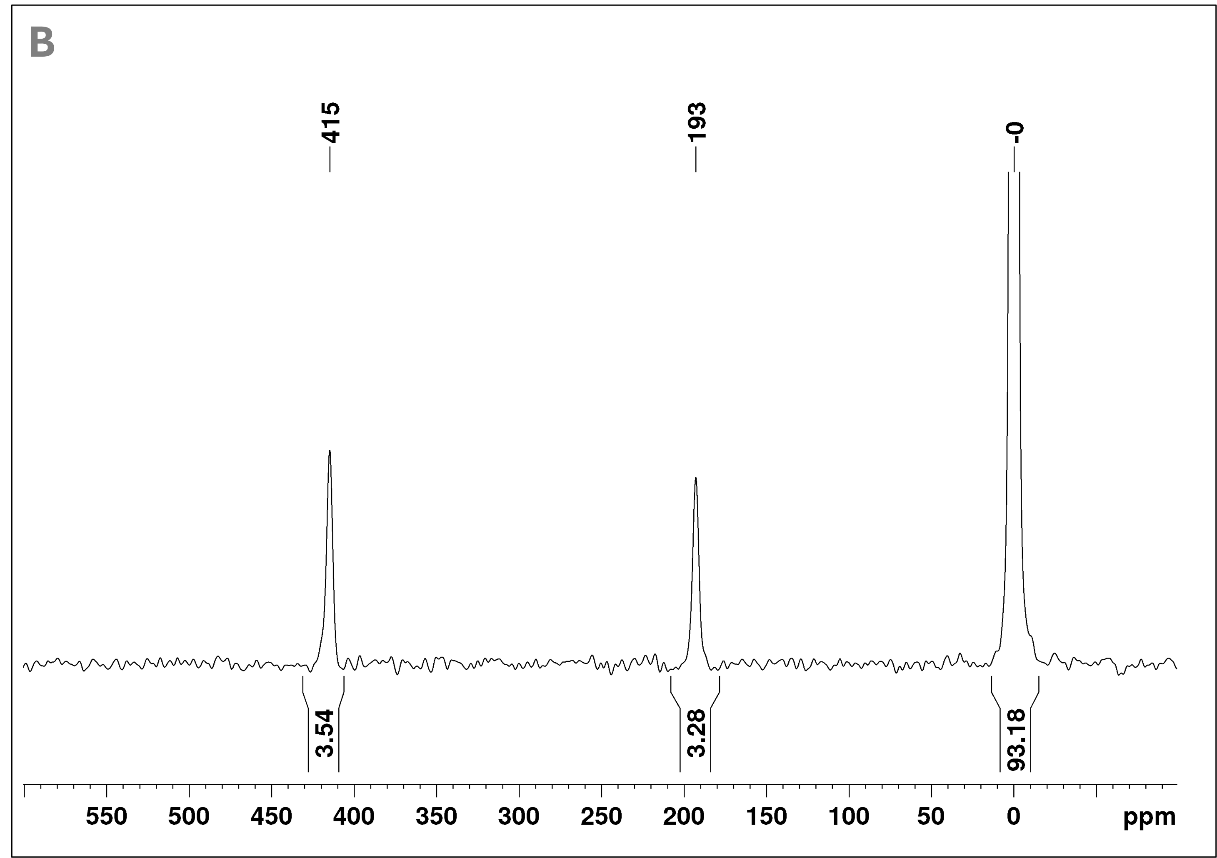
***

***Fig. S6:*** ^17^O-NMR spectrum of an equimolar mixture from NaNO_3_ (δ 415 ppm) and Na_2_CO_3_ (δ 193 ppm) in D_2_O (δ 0 ppm).
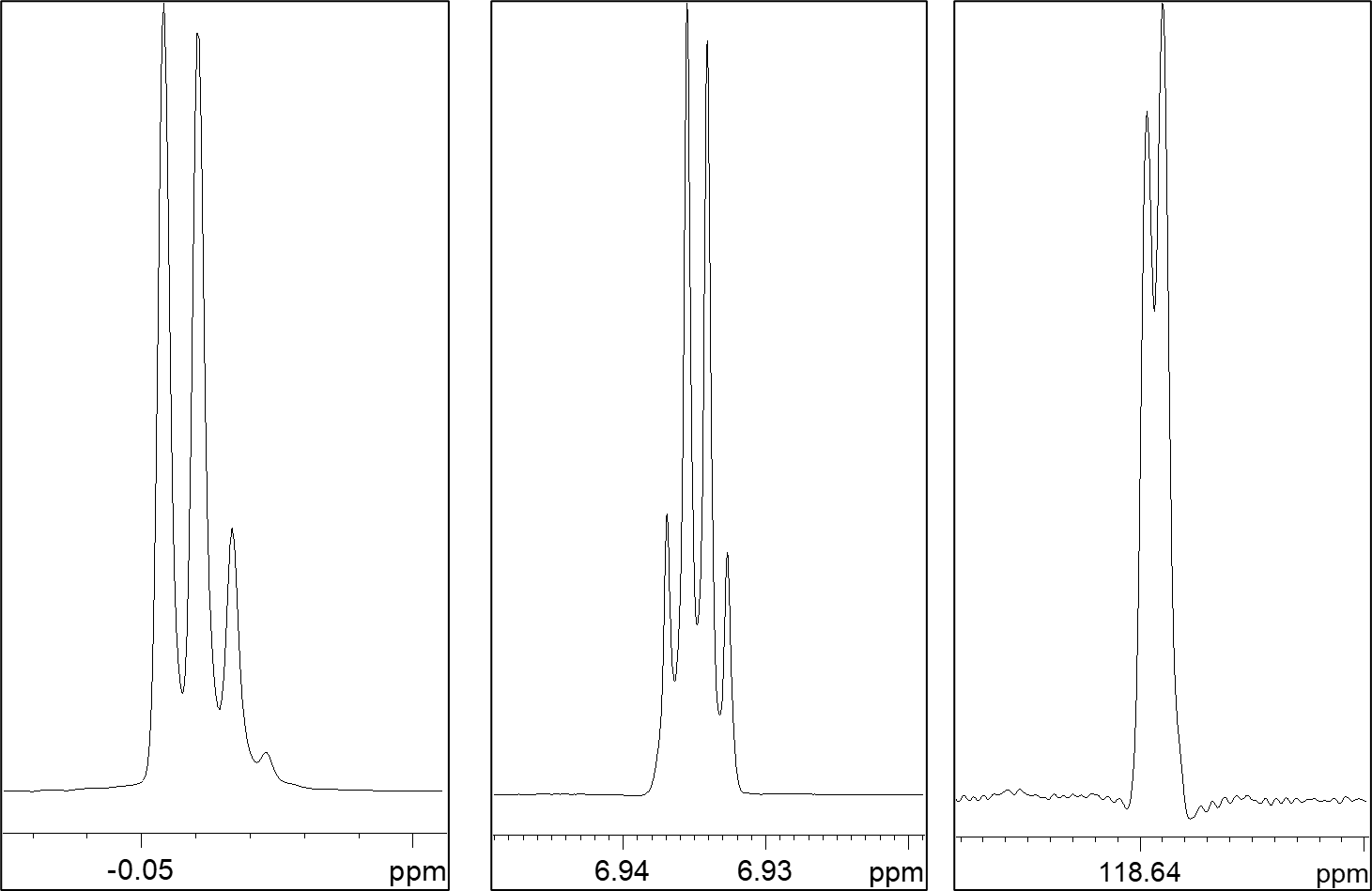


***Fig. S7:*** ^19^F-NMR of Cl_3_CF (left), ^19^F-NMR of Br_3_CF (middle) and ^13^C-NMR of Br-Phenyl ipso C atom (right). The isotope patterns are quantitatively equivalent to mass spectrometry (MS).

**Table S1.** Model mixtures of salts in aqueous solution.

|  | **NaCl [mg]** | **KBr [mg]** | **KCl [mg]** | **NaBr [mg]** | **D_2_O [ml]** |
| --- | --- | --- | --- | --- | --- |
| A1 | 30 | 150 | -- | -- | 1.0 |
| B1 | 60 | 120 | -- | -- | 1.0 |
| C1 | 90 | 90 | -- | -- | 1.0 |
| D1 | 120 | 60 | -- | -- | 1.0 |
| A2 | -- | -- | 30 | 150 | 1.0 |
| B2 | -- | -- | 60 | 120 | 1.0 |
| C2 | -- | -- | 90 | 90 | 1.0 |
| D2 | -- | -- | 120 | 60 | 1.0 |
